# Mismatch-Repair Protein Expression in High-Grade Gliomas: A Large Retrospective Multicenter Study

**DOI:** 10.3390/ijms21186716

**Published:** 2020-09-14

**Authors:** Mario Caccese, Tamara Ius, Matteo Simonelli, Matteo Fassan, Daniela Cesselli, Angelo Dipasquale, Francesco Cavallin, Marta Padovan, Alessandro Salvalaggio, Marina Paola Gardiman, Miran Skrap, Vittorina Zagonel, Giuseppe Lombardi

**Affiliations:** 1 Department of Oncology, Oncology 1, Veneto Institute of Oncology—IRCCS, 35128 Padua, Italy; marta.padovan@iov.veneto.it (M.P.); vittorina.zagonel@iov.veneto.it (V.Z.); giuseppe.lombardi@iov.veneto.it (G.L.); 2 Clinical and Experimental Oncology and Immunology PhD Program, Department of Surgery, Oncology and Gastroenterology, University of Padua, 35128 Padua, Italy; 3 Department of Neurosurgery, University Hospital of Udine, 33100 Udine, Italy; tamara.ius@gmail.com (T.I.); skrap@aoud.sanita.fvg.it (M.S.); 4 Humanitas Clinical and Research Center—IRCCS, 20089 Rozzano, Italy; matteo.simonelli@hunimed.eu (M.S.); angelo.dipasquale@cancercenter.humanitas.it (A.D.); 5 Department of Biomedical Sciences, Humanitas University, 20090 Pieve Emanuele, (Milan), Italy; 6 Surgical Pathology Unit, Department of Medicine, University of Padua, 35128 Padua, Italy; matteo.fassan@unipd.it (M.F.); marinapaola.gardiman@aopd.veneto.it (M.P.G.); 7 Department of Medicine, University of Udine, 33100 Udine, Italy; daniela.cesselli@uniud.it; 8 Independent Statistician, 36020 Solagna, Italy; cescocava@libero.it; 9 Clinica Neurologica, Department of Neuroscience, and Padova Neuroscience Center (PNC), University of Padua, 35128 Padua, Italy; salvalaggio.a@gmail.com

**Keywords:** high-grade glioma, mismatch repair, microsatellite instability, immunotherapy

## Abstract

Background: DNA mismatch repair (MMR) is a system for repairing errors in DNA replication. Cancer cells with MMR deficiency can have immunohistochemical loss of MMR protein expression leading to a hypermutable phenotype that may correlate with anti-PD1 efficacy. Scant data exist about immunohistochemical loss of MMR protein expression in high-grade gliomas (HGG). Materials and Methods: We performed a large multicenter retrospective study to investigate the frequency and the prognostic role of immunohistochemical loss of MMR protein expression in HGG patients; we nevertheless evaluated the association between this status and clinical or molecular characteristics. Immunohistochemical loss of MMR protein expression was recorded as partial or complete loss of at least 1 MMR protein. Results: We analyzed the expression of MMR proteins in tumor tissue of 355 consecutive patients. Partial and complete immunohistochemical loss of MMR proteins was found in 43/355 samples (12.1%) and among these, 15 cases (4.2%) showed a complete loss of at the least one MMR protein. Alteration of MSH2 expression was found in 55.8%, MSH6 in 46.5%, PMS2 in 34.9%, and MLH1 in 30.2%. Alteration of MMR protein expression was statistically more frequent in anaplastic gliomas, in recurrent disease, in patients treated with temozolomide, and in IDH-mut gliomas. Immunohistochemical loss of MMR proteins was not associated with survival, adjusting for clinically relevant confounders. Conclusions: MMR protein expression status did not affect survival in HGG patients. We identified clinical and molecular characteristics correlating with immunohistochemical loss of MMR proteins expression. A large study should be performed to analyze its predictive role of immune checkpoint inhibitor efficacy in these subgroups of patients.

## 1. Introduction

High-grade gliomas (grade III and IV gliomas according to 2016 WHO CNS classification [1]) represent aggressive primary brain tumors in adults; although the progress of recent years has allowed us to obtain more information about these diseases, from both a clinical and a molecular point of view, treatment options remain limited and prognosis remains poor. DNA mismatch repair (MMR) is a system to identify and repair mismatched nucleotides (erroneous insertion, deletion, and misincorporation) in order to guarantee genomic stability and integrity. Essentially, the MMR system depends on four key genes: mutS homologue 2 (MSH2), mutS homologue 6 (MSH6), mutL homologue 1 (MLH1), and post-meiotic segregation increased 2 (PMS2). These main proteins of the MMR complex are usually identified through immunohistochemical analysis in clinical settings. In the case of MMR proteins that are not expressed, dysfunctional, or inactivated, it could induce an hypermutated profile in cancer cells showing 10 to 100 times more somatic mutations than MMR-proficient cancers; this hypermutation state could lead to the generation of neoantigens that can activate the immune system and promote antitumor activity [2]. Indeed, Hodges et al. [3] demonstrated that immunohistochemical loss of at least one MMR protein expression was associated with the hypermutation profile in glioma patients. This correlation was confirmed in further recent studies [4,5,6]. Immune checkpoint inhibitors, in particular Nivolumab and Pembrolizumab (anti PD-1), were tested in several types of tumors with MMR deficiency (MMRd), showing impressive antitumor activity [7,8,9,10,11,12]; based on these results, the FDA approved the use of pembrolizumab in patients with any solid tumor with MMRd after progression from prior chemotherapy [13]. However, in HGG patients, immune checkpoint inhibitors demonstrated poor efficacy: nivolumab failed to extend survival compared to standard treatment in two phase III trials analyzing newly diagnosed MGMT (O6-Mthylguanine-DNA Methyltrasferase)-methylated and recurrent glioblastoma patients [14]; moreover, pembrolizumab showed poor results in a phase Ib trial compared to bevacizumab, in selected recurrent PD-L1 positive, glioblastoma patients [15]. In all these studies, patient selection according to MMR status or TMB (Tumor Mutational Burden) was not performed. Scant data are available on MMR complex proteins in high-grade gliomas [16], and deficit of expression of MSH6 might contribute to alkylating agent resistance and recurrence [17,18]; despite that, the role of MMRd in terms of survival in this setting of patients seems unclear. In order to improve knowledge of MMR status in high-grade gliomas and in consideration of its possible predictive role of immune checkpoint inhibitor efficacy, we performed a large multicenter study to investigate the frequency and the type of immunohistochemical loss of MMR protein expression, its association with clinical, histological, and molecular characteristics, and its correlation with survival in HGG patients.

## 2. Results

### 2.1. Patient Characteristics

Between April 2018 and May 2019, 355 consecutive high-grade glioma patients (median age 56 years; 241 males and 114 females) were enrolled. The study cohort included 315 (88.7%) grade IV glioblastoma and 40 (11.3%) grade III glioma (30 anaplastic astrocytoma, 2 anaplastic ependymoma, and 8 anaplastic oligodendroglioma). Patient characteristics (overall and stratified by grade III and IV glioma) are reported in Table 1. Eastern Cooperative Group (ECOG) performance status (PS) at diagnosis was ECOG 0-1 in 87.3% of patients and radical surgery was performed in 70.4% of cases. Molecular analyses were done on the primary tumor in 85% of cases and in 15% on recurrent tumors hence; MMR status analysis was performed after a first line of concomitant temozolomide and radiation therapy. MGMT proved methylated in 161/300 patients (53.7%) and IDH (isocitrate dehydrogenase) was of wild-type in 267/315 (84.4%). IHC (Immunohistochemistry) analysis of PD-L1 expression was performed in 150 samples, with no expression of PD-L1 in 66% of them and PD-L1 expression >0% in 34% of them.

### 2.2. Mismatch Repair Status

**Figure 1:**
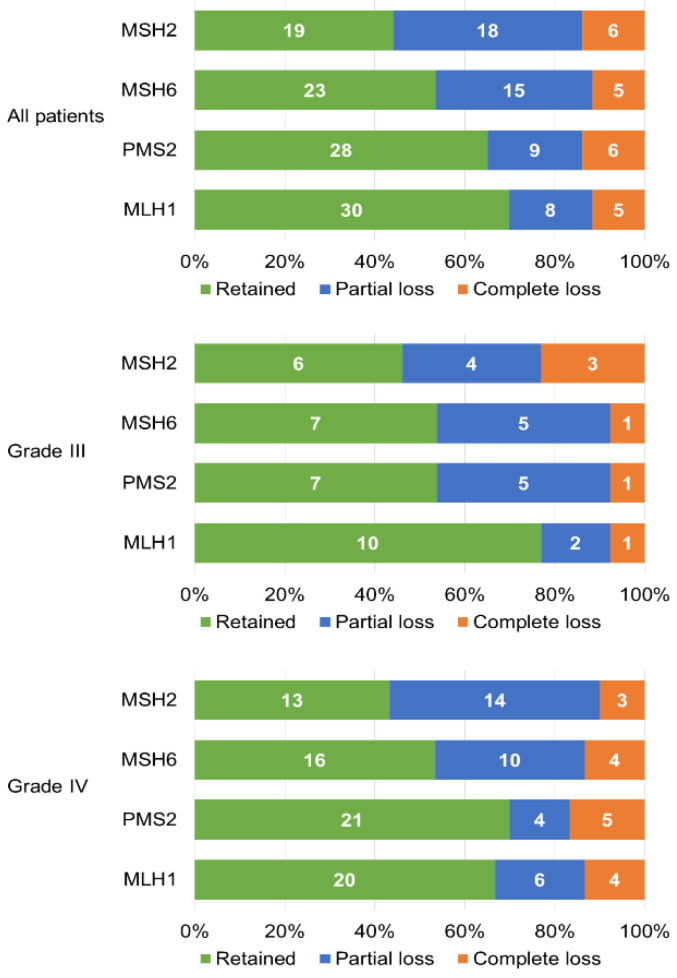
Characteristics of immunohistochemical expression of MMR (Mismatch Repair) proteins in our cases.

IHC analysis identified immunohistochemical loss of MMR protein expression (partial or complete) in 43/355 (12.1%) patients; among these, 15/355 showed a complete immunohistochemical loss of at least one MMR protein (4.2%). Both MSH2 and MSH6 were proven lost in 14/43 (33%) patients (11/43 and 3/43 with partial and complete loss, respectively), while both PMS2 and MLH1 were lost in 10/43 (23%) patients (5/43 with partial loss and 5/43 with complete loss). Partial or complete loss of at least one MMR protein was found in 13/40 (32.5%) grade III patients and 30/315 (9.5%) grade IV patients. Mismatch repair status is summarized in Figure 1.

### 2.3. Mismatch Repair Deficiency and Clinical/Molecular Characteristics

**Figure 2:**
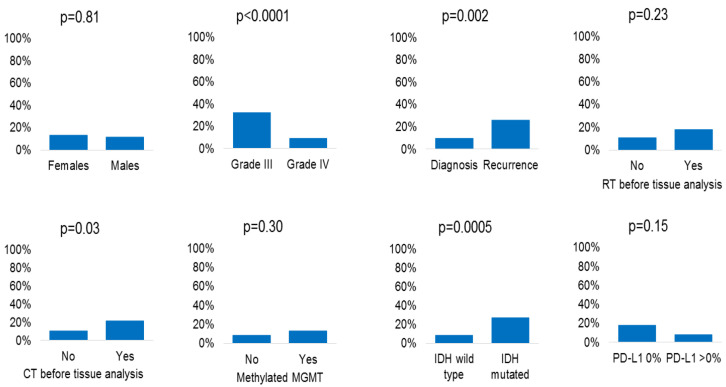
Association between immunohistochemical loss of MMR protein expression and clinical/molecular characteristics.

The association between immunohistochemical loss of MMR protein expression and clinical/molecular characteristics is shown in Figure 2 (numerical data in Table 2). This molecular alteration was more frequent in grade III vs. IV gliomas (*p* < 0.0001), in tissue samples obtained at recurrence (*p* = 0.002) or after chemotherapy (*p* = 0.03), and in patients with mutated IDH (*p* = 0.0005). No statistically significant association was found between immunohistochemical loss of MMR protein expression and sex (*p* = 0.81), radiotherapy before tissue analysis (*p* = 0.23), MGMT methylation status (*p* = 0.30), and PD-L1 expression (*p* = 0.15). In patients with immunohistochemical complete loss of MMR protein expression (15/355), there was a statistically significant correlation between the presence of this alteration and the chemotherapy (*p* = 0.002) and radiotherapy (*p* = 0.02) received before the analysis, as well as disease recurrence (*p* = 0.02) (Supplementary Materials Figure S2). Sub-analysis stratified by grade III/IV is reported in Table 2. The association between immunohistochemical loss of MMR proteins and tissue samples obtained at recurrence was confirmed (*p* = 0.003) in grade IV patients, while the other associations showed a trend towards statistical significance. The small number of grade III patients did not allow us to identify any statistically significant association.

### 2.4. Loss of Mismatch Repair Proteins and Survival

**Figure 3:**
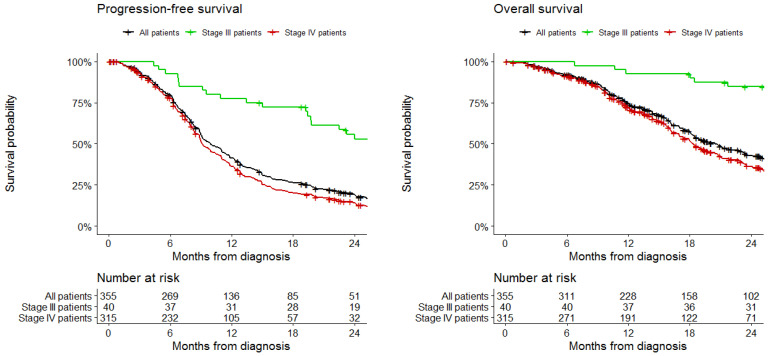
Progression-free survival and overall survival in the study cohort, stratified by grade.

Median follow-up was 16 months (IQR 16–20). Two-year OS was 42% in the study cohort (82% in grade III patients and 36% in grade IV patients). Median OS was 20 months (95%CI 18 to 23 months), by grade: 94 months (95%CI 76 to NA months) in grade III patients and 18 months (95%CI 17 to 21 months) in grade IV patients. Two-year PFS was 19% in the study cohort (53% in grade III patients and 14% in grade IV patients). Median PFS was 10 months (95%CI 9 to 11 months), by grade: 26 months (95%CI, 20 to 45 months) in grade III patients and 9 months (95%CI 9 to 10 months) in grade IV patients. OS and PFS data are shown in Figure 3. At multivariable analysis (Table 3), immunohistochemical loss of MMR proteins expression was not associated with PFS (HR 1.05; 95%CI 0.55 to 2.01; *p* = 0.89) or OS (HR 0.72; 95%CI 0.28 to 1.86; *p* = 0.50). Impaired PFS was associated with wild-type IDH (HR 4.73, 95% CI 1.54 to 14.48; *p* = 0.007), while impaired OS was associated with poor performance status (HR 4.87, 95% CI 1.86 to 12.79; *p* = 0.001) and unmethylated MGMT (HR 2.03, 95% CI 1.01 to 4.08; *p* = 0.04).

### 2.5. Survival in Patients with Immunohistochemical Loss of MMR Proteins Expression

**Figure 4:**
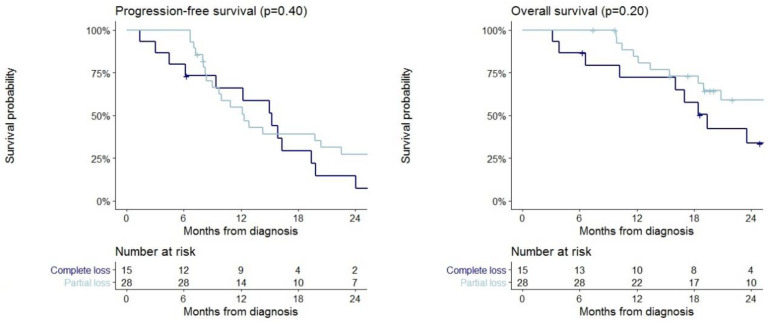
Progression-free survival and overall survival in patients with complete (*n* = 15) and partial (*n* = 28) loss of MMR protein expression.

The 43 patients with immunohistochemical loss of MMR protein expression were divided into 15 and 28 patients with complete or partial loss of at least one MMR protein expression, respectively. No statistically significant difference between the two groups in terms of PFS and OS was demonstrated. Median PFS was 15 months (95% CI 9–24) in patients with complete loss of MMR protein expression and 12 months (95% CI 10–26) for those with partial loss expression. Median OS was 19 months (95% CI 16-NA) in patients with complete loss of MMR protein expression and 76 months (95% CI 19-NA) for those with partial loss of MMR protein expression. Survival curves stratified by complete and partial MMR expression are reported in Figure 4.

## 3. Discussion

In this retrospective and multicenter study, we identified a small group of high-grade glioma patients, with immunohistochemical loss of mismatch repair protein expression: 12% of screened patients reported a “partial” or “total” loss of expression of one or more MMR complex proteins (MSH2, MSH6, MLH1, and PMS2). We found a higher probability of immunohistochemical loss of MMR proteins in grade III than in grade IV gliomas, at recurrence, in patients receiving temozolomide therapy and in IDH-mut gliomas. In patients with immunohistochemical complete loss of MMR protein expression, a statistically significant correlation between the presence of this alteration and the chemotherapy and radiotherapy received before the analysis, as well as disease recurrence, was found. As already reported in the literature [19], grade III gliomas are different entities compared to grade IV tumors from several viewpoints; in our population, 72% of grade III tumors reported an IDH mutation compared to only 8% in glioblastoma patients. In particular, the presence of mutated IDH can affect epigenetic alterations and lead to a higher probability of alteration of MMR protein expression in grade 3 gliomas. Moreover, immunohistochemical loss of MMR protein expression may be useful to detect hypermutated cancers as demonstrated by McCord et al. [6]; in this study, the authors showed that IHC loss of MMR proteins can detect hypermutated gliomas with high sensitivity and specificity; yet, in this study, 9 out of 100 gliomas were hypermutated and 8 out of 9 had complete loss of expression of at least one MMR protein. All hypermutated patients had previously received temozolomide. Even the tumor microenvironment can prove different between grade 2 and grade 3 gliomas; Pinton et al. [20] showed that in the glioblastoma microenvironment, there are more macrophages with immunosuppressive activity compared to grade II–III gliomas mostly consisting of microglia with a limited or absent immunosuppressive activity. Knowledge of the predictors of immunotherapy efficacy is extremely important to identify the subgroup of patients responding to this treatment; hence, the immunohistochemical loss of MMR protein expression could be a valid predictor of checkpoint inhibitor efficacy in gliomas. Noteworthy, a large prospective study should be performed in order to validate this biomarker and the role of partial and total immunohistochemical loss of MMR protein expression. In our study, in 55 cases (15%), IHC analysis was performed on tissue obtained after temozolomide; in these patients, we showed a higher probability of immunohistochemical loss of MMR protein expression compared to the cases analyzed before temozolomide therapy. As described in the literature, temozolomide and other alkylating agents can cause alterations in different genes with consequent acquisition of a hypermutated phenotype that can also determine temozolomide resistance. In fact, alkylating agents can alter MMR protein expression and lead to hypermutated genome, with a mechanism independent from microsatellite instability [17]. The specific mechanism that determines this phenomenon is still unclear but it is hypothesized that temozolomide may exert a selective pressure on tumor clones specifically targeting for MMR loss (in particular, MSH6) and that some post-translational changes in the subcellular localization of MMR heterodimers, in response to the alkylating action, may be partly responsible for loss of MMR proteins expression [17]. Indeed, we performed an MSI analysis (assessed using the Titano Kit, Diatech Pharmacogenetics, that analyzes six poly-A microsatellite BAT25, BAT26, BAT40, NR21, NR24, and TGFβRII and four dinucleotide markers D2S123, D17S250, D5S346, and D18S58) on a small group of patients (12/355), and none showed the presence of microsatellite instability. To date, it is unclear whether hypermutations induced by temozolomide treatment are immunogenic or not; moreover, we recorded a deficit of expression of one or more MMR proteins in 31 patients (9%), in which IHC analysis were performed on primary diagnosis and microsatellite was stable; these cases remain unclear and, likely, the loss of MMR protein expression can be independent of microsatellite status, as already reported in other papers [4]. The correlation shown in our study between immunohistochemical loss of MMR protein expression and recurrent gliomas is in line with previous papers demonstrating a different expression profile of MMR proteins between diagnosis and recurrence disease. Indeed, Felsberg et al. [21], comparing the expression of MSH2, MSH6, MLH1, and PMS2 in primary and recurrent glioblastoma, demonstrated a significantly lower MSH2, MSH6, and PMS2 expression in recurrent GBM. Nevertheless, Cahill and colleagues [17] detected MSH6 mutations in 3 of 14 recurrent brain tumors, but no mutation of MSH6 in 40 pretreatment GBM, suggesting that loss of MSH6 expression might set in during temozolomide treatment. Another work showed that MLH1 expression was significantly reduced at recurrence compared to the original tumor [16].

In summary, we identified, in this study, a population of patients with high-grade glioma with a higher probability of loss of MMR protein expression. This molecular alteration seems to be more present in grade III gliomas (*p* < 0.0001), in the tissue analyzed at relapse (*p* = 0.002), and after receiving chemotherapy (*p* = 0.03) as well as in IDH-mut patients (*p* = 0.0005). These data could allow us to identify patients who could benefit from immune checkpoint inhibitors treatment. In a recent work, Lombardi et al. [22], demonstrated, in a small cohort of patients with relapsed glioblastoma, that loss of MMR protein expression cannot be considered a predictor of response to pembrolizumab [23]. These data should be confirmed, also in light of the analyses performed by our study, in a larger population and after careful selection of patients.

## 4. Materials and Methods

### 4.1. Study Design

This is a multicenter and retrospective study evaluating MMR status in all consecutive patients treated for grade III–IV glioma between April 2018 and May 2019. The study was approved by the local ethics committee (number 8/2019) and complied with International Ethical Guidelines for Biomedical Research Involving Human Subjects, good clinical practice guidelines, and the Declaration of Helsinki. The participating centers were the Veneto Institute of Oncology (Padua), the Humanitas Cancer Center (Rozzano, Milan), and the Santa Maria della Misericordia University Hospital (Udine).

### 4.2. Patients

All consecutive patients treated for grade III–IV glioma at the participating centers between April 2018 and May 2019 were evaluated for inclusion. Inclusion criteria were at least 18 years of age, histologically confirmed diagnosis of grade III or IV glioma, and tumor tissue available for immunohistochemical analysis resulting from the first or subsequent surgery.

### 4.3. Procedures

After obtaining written informed consent for each patient, we retrospectively collected and analyzed the tumor tissue with high-grade glioma diagnosis (grade III and IV according to 2017 WHO Classification of Central Nervous System Tumors), undergoing one or more neurosurgical interventions and treated at the participating centers. We evaluated the expression of four major proteins of the MMR complex (MSH2, MHH6, MLH1, and PMS2) and defined immunohistochemical loss of MMR proteins as partial or complete loss of the expression of one or more MMR proteins.

The mismatch repair status was determined by examining the loss of protein expression by immunohistochemistry of four MMR enzymes (MLH1, PMS2, MSH2, and MSH6; Dako, Glostrup, Denmark); expressions of these four proteins were considered “present” (+/+) in the case of unequivocal nuclear labeling in tumor cells with staining intensity comparable to that of internal control, “partial loss” (+/−) in the case of visible nuclear labeling in tumor cells, but with an intensity weaker than the internal control or only comparable to the intensity of the inert stromal cells, and “complete loss” (−/−) in the case of no visible nuclear labeling in tumor cells. MGMT promoter methylation status was analyzed by pyrosequencing technology using a commercially available kit (MGMT plus^®^, Diatech Pharmacogenetics, Jesi, Italy) according to manufacturer’s instructions on a PyroMark TM Q96 ID system (Qiagen, Hilden, Germany) with PyroMark CpG (Qiagen) software.

Immunohistochemistry with anti-PD-L1 primary antibodies was performed using the BOND-MAX system (Leica Biosystems). Immunostainings were jointly assessed by two pathologists.

### 4.4. Data Collection

Clinical, histological, and molecular characteristics of included patients were collected. Information about the time of surgery and the type of chemotherapy and/or radiotherapy treatment was also collected. Progression-free survival was calculated from the date of diagnosis to the date of the last follow-up visit or the date of radiological progression according to RANO (Response Assessment in Neuro-Oncology) criteria. Overall survival was calculated from the date of diagnosis to the date of the last follow-up visit or the date of the death.

### 4.5. Statistical Analysis

Data were presented as median with interquartile range (IQR) or absolute frequency with percentage (%). The association between immunohistochemical loss of MMR proteins and clinical/molecular characteristics was evaluated using the Chi-square test and Fisher’s exact test. Survival curves were estimated with the Kaplan–Meier method and compared using a log-rank test. Median survival (with 95% confidence interval) was also calculated. The upper-bound of the confidence interval for overall survival could not be calculated in some subgroups and was indicated as “not available” (NA). Cox regression models were estimated to evaluate the effect of immunohistochemical loss of MMR proteins expression on overall survival (OS) and progression-free survival (PFS), adjusting for clinically relevant confounders. Effect sizes were expressed as hazard ratio (HR) with 95% confidence interval (CI). All tests were 2-sided and a *p*-value less than 0.05 was considered statistically significant. Data analysis was performed using R 4.0 (R Foundation for Statistical Computing, Vienna, Austria) [23].

## 5. Conclusions

In conclusion, we identified a specific subgroup of high-grade glioma patients with immunohistochemical loss of MMR protein expression. We showed no correlation between MMR protein expression status and survival. However, this characteristic could be a valid biomarker of checkpoint inhibitor efficacy, but a larger study is needed to analyze and confirm this hypothesis.

## Figures and Tables

**Table 1:** Patient characteristics.

	All Patients	Patients with Grade III	Patients with Grade IV
No. of patients	355	40	315
Age at diagnosis, years ^a^	56 (45–65)	35 (31–46)	58 (48–66)
Females	114 (32.1)	14 (35.0)	100 (31.7)
Males	241 (67.9)	26 (65.0)	215 (68.3)
Grade: ^b^		40 (11.3)	315 (88.7)
PS:			
0	190 (53.5)	22 (55.0)	168 (53.3)
1	120 (33.8)	13 (32.5)	107 (34.0)
2	39 (11.0)	5 (12.5)	34 (10.8)
3	6 (1.7)	0 (0.0)	6 (1.9)
Surgery			
Non-radical	105 (29.6)	10 (25.0)	95 (30.2)
Radical	250 (70.4)	30 (75.0)	220 (69.8)
Analyzed tissue:			
Diagnosis	301 (84.8)	30 (75.0)	271 (86.0)
Recurrence	54 (15.2)	10 (25.0)	44 (14.0)
MGMT status: ^c^			
Unmethylated	139 (46.3)	2 (9.1)	137 (493)
Methylated	161 (53.7)	20 (90.9)	1412 (50.7)
IDH status: ^d^			
Wild type	267 (84.8)	9 (29.2)	258 (91.2)
Mutated	48 (15.2)	23 (71.8)	25 (8.8)
PD-L1 expression: ^e^			
0%	99 (66.0)	19 (100.0)	80 (61.1)
>0%	51 (34.0)	0 (0.0)	51 (38.9)
MMR protein expression:			
Retained	312 (87.9)	27 (67.5)	285 (90.5)
Partial loss	28 (7.9)	9 (22.5)	19 (6.0)
Complete loss	15 (4.2)	4 (10.0)	11 (3.5)

Data expressed as n (%) or ^a^ median (IQR). ^b^ Grade III included 31 anaplastic astrocytoma, 2 Anaplastic ependymoma, 1 anaplastic ganglioneuronal tumor, and 6 anaplastic oligodendroglioma, while all grade IV were glioblastoma. Data not available in ^c^ 55, ^d^ 40, and ^e^ 205 patients.

**Table 2:** Association between immunohistochemical loss of MMR protein expression and clinical/molecular characteristics.

	All Patients			Patients with Grade III			Patients with Grade IV		
	Alteration of MMR Protein Expression: n/N (%)	*p*-value	Partial:Complete Loss of MMR Protein Expression: n:n	Alteration of MMR Protein Expression: n/N (%)	*p*-Value	Partial:Complete Loss of MMR Protein Expression: n:n	Alteration of MMR Protein Expression: n/N (%)	*p*-Value	Partial:Complete Loss of MMR Protein Expression: n:n
No.	43/355 (12.1)	-	28:15	13/40 (32.5)	-	9:4	30/315 (9.5)	-	19:11
Females	15/114 (13.2)	0.81	12:3	7/14 (50.0)	0.17	6.1	8/100 (8.0)	0.67	6:2
Males	28/241 (11.6)		16:12	6/26 (23.1)		3:3	22/215 (10.2)		13:9
Grade:		<0.0001		-	-	-	-	-	-
III	13/40 (32.5)		9:4						
IV	30/315 (9.5)		19.11						
Analyzed tissue at:		0.002			0.70			0.003	
Diagnosis	29/301 (9.6)		20:9	9/30 (30.0)		7:2	20/271 (7.4)		13:7
Recurrence	14/54 (25.9)		8:6	4/10 (40.0)		2.2	10/44 (22.7)		6:4
RT before tissue analysis:		0.23			0.99			0.25	
No	33/299 (11.0)		24:9	10/30 (33.3)		9:1	23/269 (8.6)		15:8
Yes	10/56 (17.9)		4:6	3/10 (30.0)		0:3	7/46 (15.2)		4:3
CT before tissue analysis:		0.03			0.70			0.08	
No	31/300 (10.3)		23:8	9/30 (30.0)		8:1	22/270 (8.1)		15:7
Yes	12/55 (21.8)		5:7	4/10 (40.0)		1:3	8/45 (17.8)		4.4
MGMT status:		0.30			0.99			0.90	
Unmethylated	12/139 (8.6)		5:7	0/2 (0.0)		0:0	12/137 (8.8)		5:7
Methylated	21/161 (13.0)		15:6	7/20 (35.0)		5.2	14/141 (9.9)		10:4
IDH status:		0.0005			0.99			0.09	
Wild type	23/267 (8.6)		13:10	3/9 (33.3)		2.1	20/258 (7.8)		11:9
Mutated	13/48 (27.1)		8:5	8/23 (34.8)		5:3	5/25 (20.0)		3:2
PD-L1 expression:		0.15			NA			0.77	
0%	18/99 (18.2)		12:6	9/19 (47.7)		7:2	9/80 (11.3)		5:4
>0%	4/51 (7.8)		3.1	0/0		2:2	4/51 (7.8)		3:1

**Table 3:** Multivariable analysis of progression-free survival and overall survival.

	Progression-Free Survival		Overall Survival	
	Hazard Ratio (95% CI)	*p*-Value	Hazard Ratio (95% CI)	*p*-Value
Immunohistochemical loss of MMR protein expression:		0.89		0.50
No	Reference		Reference	
Yes	1.05 (0.55 to 2.01)		0.72 (0.28 to 1.86)	
Age, years	1.00 (0.98 to 1.02)	0.71	1.01 (0.98 to 1.03)	0.58
Grade:		0.22		0.09
III	Reference		Reference	
IV	2.09 (0.64 to 6.79)		4.95 (0.76 to 32.22)	
Surgery:		0.10		0.48
Radical	Reference		Reference	
Non-radical	1.66 (0.90 to 3.05)		1.34 (0.59 to 3.05)	
PS:				
0	Reference		Reference	
1	1.38 (0.82 to 2.31)	0.22	1.83 (0.85 to 3.92)	0.12
2–3	1.61 (0.85 to 3.05)	0.15	4.87 (1.86 to 12.79)	0.001
MGMT status:		0.14		0.04
Unmethylated	1.41 (0.90 to 2.23)		2.03 (1.01 to 4.08)	
Methylated	Reference		Reference	
IDH status:		0.007		0.23
Wild type	4.73 (1.54 to 14.48)		2.99 (0.51 to 17.51)	
Mutated	Reference		Reference	

## Supplementary Materials

The following are available online at https://www.mdpi.com/1422-0067/21/18/6716/s1, Supplementary Figure S1. Association between immunohistochemical PARTIAL LOSS of MMR protein expression and clinical/molecular characteristics. Supplementary Figure S2. Association between immunohistochemical COMPLETE LOSS of MMR protein expression and clinical / molecular characteristics
