# Supplementary material for: Mismatch-Repair Protein Expression in High-Grade Gliomas: A Large Retrospective Multicenter Study
Source: Int J Mol Sci. 2020 Sep 14;21(18):6716. doi: 10.3390/ijms21186716 (PMC7555820; doi:10.3390/ijms21186716)

Supplementary Figure 1. Association between immunohistochemical PARTIAL LOSS of MMR protein expression and clinical / molecular characteristics

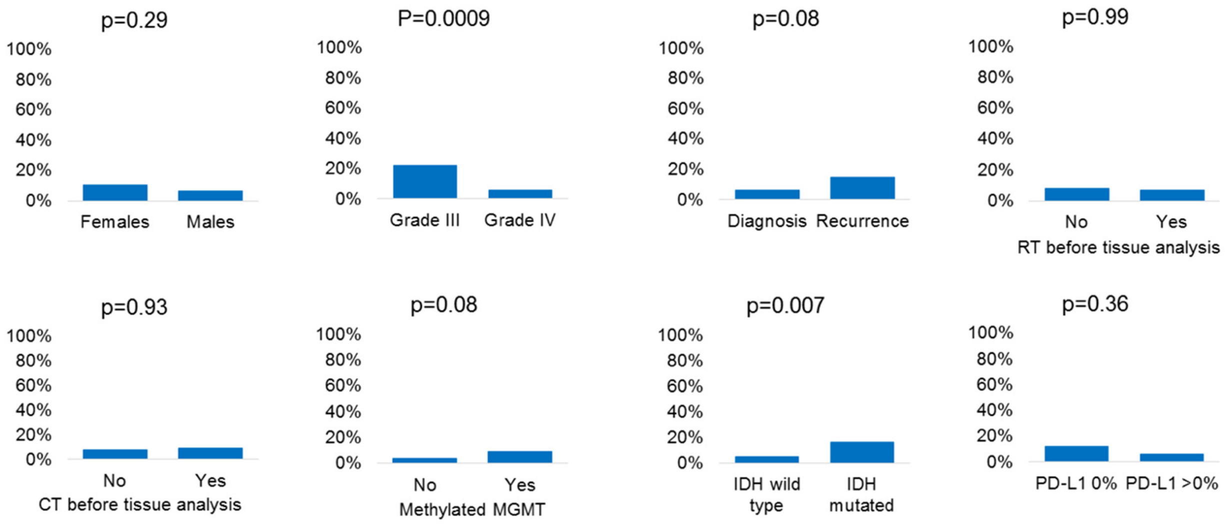

Supplementary Figure 2. Association between immunohistochemical COMPLETE LOSS of MMR protein expression and clinical / molecular characteristics

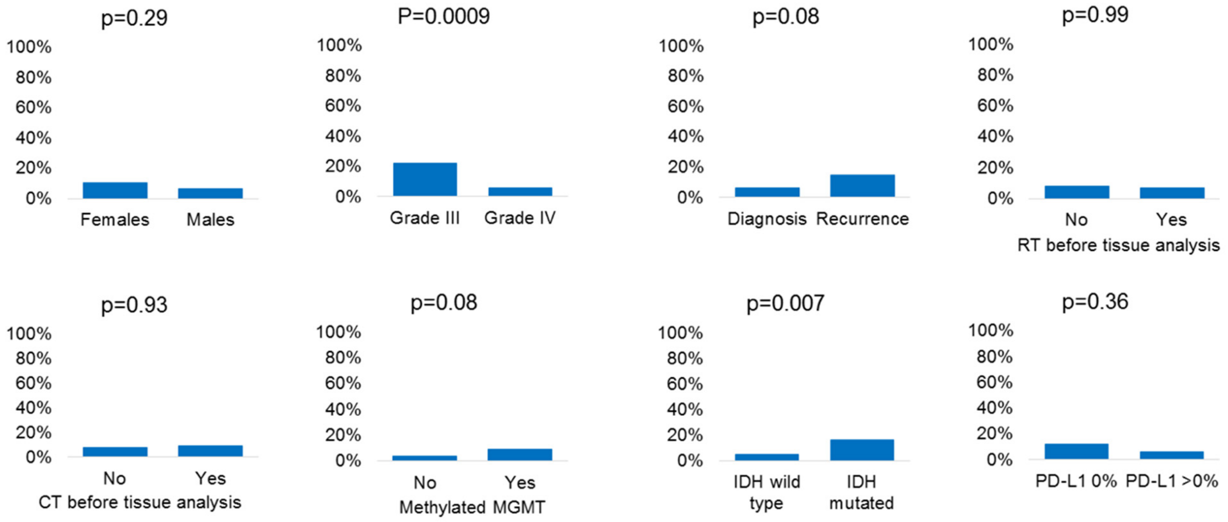

Supplement: Supplementary file 1 [file ijms-21-06716-s001.pdf]
